# Effect of Supplementation with *Curcuma longa* and *Rosmarinus officinalis* Extract Mixture on Acute Phase Protein, Cathelicidin, Defensin and Cytolytic Protein Gene Expression in the Livers of Young Castrated Polish White Improved Bucks

**DOI:** 10.3390/genes14101932

**Published:** 2023-10-12

**Authors:** Daria M. Urbańska, Marek Pawlik, Agnieszka Korwin-Kossakowska, Michał Czopowicz, Karolina Rutkowska, Ewelina Kawecka-Grochocka, Marcin Mickiewicz, Jarosław Kaba, Emilia Bagnicka

**Affiliations:** 1 Department of Animal Improvement and Nutrigenomics, Institute of Genetics and Animal Breeding Polish Academy of Sciences, Postepu 36A Str., 05-552 Jastrzębiec, Poland; agnieszka.kossakowska@gmail.com (A.K.-K.); e.bagnicka@igbzpan.pl (E.B.); 2 Department of Neurotoxicology, Mossakowski Medical Research Institute Polish Academy of Sciences, Pawińskiego 5 Str., 02-106 Warsaw, Poland; pawlik.marek@gmail.com; 3 Division of Veterinary Epidemiology and Economics, Institute of Veterinary Medicine, Warsaw University of Life Sciences-SGGW, Nowoursynowska Str. 159c, 02-776 Warsaw, Poland; michal_czopowicz@sggw.edu.pl (M.C.); marcin_mickiewicz@sggw.edu.pl (M.M.); jaroslaw_kaba@sggw.edu.pl (J.K.); 4 Department of Medical Genetics, Medical University of Warsaw, Pawińskiego 3c Str., 02-106 Warsaw, Poland; karolina.rutkowska@wum.edu.pl

**Keywords:** hepatic immune system, mRNA level, turmeric–rosemary mixture, dietary supplement, male kid

## Abstract

Goats are an excellent animal model for research on some physiological and pathophysiological processes in humans. The search for supplements that prevent homeostasis disorders and strengthen the immune system is necessary to reduce the risk of many diseases in both humans and animals. The aim of the study was to analyze the effect of supplementation with a mixture of dried extracts of *Curcuma longa* and *Rosmarinus officinalis* on the expression of acute-phase protein (*SAA*, *HP*, *CRP*, *LALBA*, *AGP*, *CP*, *FGA*, *FGB*, and *FGG*), cathelicidin (*BAC5*, *BAC7.5*, *BAC3.4*, *MAP28*, *MAP34*, and *HEPC*), beta-defensin-1 (*GBD1*, *DEFB1*), and beta-defensin-2, and cytolytic protein (*LIZ* and *LF*) genes in the livers of young castrated bucks of the Polish White Improved breed. The higher expression of *LF* in the control group suggests that it is important for the first line of hepatic immune defense and its expression is downregulated by the mixture of turmeric and rosemary extracts; thus, the spice–herb mixture mutes its activity. The lower expression of *FGB* and the higher expression of *BAC5* genes in the livers of healthy, young castrated bucks who were administered the supplement suggest the silencing effects of the mixture on the acute-phase response and the stimulating effect on the antimicrobial activity of the immune system.

## 1. Introduction

As meat is an important component of the human diet because it contains essential nutrients and food safety is a fundamental issue, meat should be obtained from healthy animals [1]. One of the indicators of the health state of the animal is the functioning of its liver. It is one of the most regenerative mammalian tissues. The liver participates in digestion, splanchnic tissue regulation, detoxification, fluid and electrolyte balance, hemostasis, and immunological defense activities. It can restore its own functions by replicating existing cells. Under normal conditions, the hepatocytes may persist for weeks to months without dividing [2]. The liver is an important organ that plays a key role in pathogen detection during infection. It is able to induce a rapid and strong immune response due to the presence of a large number of phagocytic cells [3].

To prevent homeostasis disturbances and strengthen the immune system, it is necessary to reduce the risk of various diseases through appropriate supplementation. Polyphenols, found in fruits, vegetables, grains, tea, essential oils, spices, and herbs, are natural antioxidants and chemo-preventers. These compounds also display anti-inflammatory effects and interfere with oxidative stress signaling [4]. One of the spices containing functional substances is *Curcuma longa* (turmeric), rich in the polyphenol curcumin (77%), which has anti-infective, anti-inflammatory, antioxidant, hepatoprotective, and anti-cancer effects [5]. Curcumin, like other phenolic compounds, in low doses can promote the formation of hydroxyl radicals. However, in high doses, it protects against them [6]. Another plant rich in polyphenols is *Rosmarinus officinalis* (rosemary), which contains, among others, carnosol, carnosic acid, methyl carnosol, and rosemary acid. Rosemary exhibits antioxidant properties due to its iron chelating ability, which removes free peroxide radicals and inhibits lipid peroxidation. In addition, it has anti-cancerogenic and tumor-reducing effects [7].

Acute-phase proteins (APPs) are a crucial component of the immune system and belong to a large, diverse group of proteins. The restoration of homeostasis is the primary function of APPs. They activate the complement system, opsonization, agglutination, and the release of lysosomal enzymes; reduce tissue damage caused by bacteria; and enhance chemotaxis. They are mainly produced in the liver during infections, inflammations, tumors, and stress [8], and they are classified as positive or negative APPs depending on their concentration in pathological conditions. In goats, the positive APP group includes serum amyloid (SAA), haptoglobin (Hp), ceruloplasmin (Cp), fibrinogen (Fb), C-reactive protein (CRP), and α1-acid glycoprotein (AGP). APP concentrations increase rapidly 24–48 h after infection. Only lactalbumin (LALBA) is a negative APP, and its concentration usually decreases during infection [9].

Moreover, multifunctional antimicrobial peptides, called host defense peptides (HDPs) or antimicrobial peptides (AMPs), take part in the body’s defense, destroying the cell membranes of microorganisms. They exhibit direct action against bacteria, both Gram-positive and Gram-negative ones, enveloped viruses, fungi, parasites, and cancerous cells. However, the extent of action depends on their concentration at the site of action [10]. Defensins and cathelicidins are the most numerous and best known of the multifunctional antimicrobial peptides [11,12]. Regardless of their location or structure, they are built of about 60 amino acid residues. They are hydrophobic and/or amphipathic and cationic, and they act within cell membranes. Cathelicidins and defensins occur among others in the respiratory and digestive tracts, muscle, bone marrow, and the thymus, as well as in many organs (such as the spleen, the brain, the liver, kidney, and the heart), in the skin, on mucosal surfaces, and in other epithelia [13].

The highest concentrations of defensins are in the storage organelles of leukocytes. To date, in the goat genome, 50 beta-defensin genes have been identified. However, only the beta-defensin 1 (*GBD-1, DEFB1*) and beta-defensin 2 genes have been studied in more depth [12]. Hepcidin (HEPC) is also classified as an AMP, namely defensin, with multiple immune activities despite having four cysteine (disulfide) bridges instead of three (as in classical defensins). The main site of HEPC production is the liver, but it is also detected in the kidneys, pancreatic beta cells, adipose tissue, heart, and body fluids [14]. It has direct antiviral and antimicrobial activity, but HEPC also takes part in iron metabolism. During infections, HEPC binds iron, reducing extracellular iron availability for iron-dependent bacteria. Consequently, it limits the use of iron by pathogenic bacteria acting indirectly against pathogens [15].

Cathelicidins are important species-specific agents participating in the innate immune defense as well. They present chemotactic activity, regulate inflammation, and exert antimicrobial activity, including activity against bacteria and enveloped viruses. In goats, five cathelicidins have been identified so far, namely capra hircus bacetnecin 3.4 (ChBAC3.4), bactenecin 5/cathelicidin 2 (BAC5), bactenecin 7.5/cathelicidin 3 (BAC7.5), cathelicidin 6 (MAP28), and cathelicidin 7 (MAP34) [16].

The crucial components in the formation of immune response are pattern recognition receptors (PRRs). They bind to pathogen-associated molecular patterns (PAMPs) present in different microbial agents [17]. Lactoferrin (LF) is one of the molecules recognizing PAMPs. It is a multifunctional iron-binding glycoprotein. Moreover, LF responds to the presence of LPS and bacterial unmethylated CpG DNA. LF is believed to participate in adaptive and innate immunities. It is released from neutrophils immediately or within hours after exposure to an antigen into the blood and inflamed tissues. During immune response, LF modulates the migration, maturation, and functions of immune cells by interacting with proteoglycans and receptors of innate immune cells such as NK cells, neutrophils, macrophages [18].

An important non-specific antimicrobial factor is lysozyme (LIZ). It is present in milk, many mucosal secretions (such as tears, saliva, and intestinal mucus), liver granulomas, Kupffer cells, and animal tissues. LIZ modulates the inflammatory response as well as binds important pathogenic agents, such as lipopolysaccharide (LPS), lipoteichoic acid (LTA), and peptidoglycan, present in the cell walls of Gram-negative and Gram-positive bacteria. Consequently, it prevents the interaction of any toxic bacterial component with receptors on intestinal epithelial cells and intestinal macrophages. It suppresses the production of tumor necrosis factor α (TNF-α), increases the expression of anti-inflammatory cytokines, and exerts immunomodulatory effects on neutrophils [19].

We assumed that the mixture of dried curcumin and rosemary extracts influences the innate immunity of the young castrated bucks, contributing to the maintenance of homeostasis. Thus, the aim of this study was to analyze the effect of supplementation with a mixture of dried extracts from *C. longa* and *R. officinalis* on the expression of acute phase protein (*SAA*, *HP*, *CRP*, *LALBA*, *AGP*, *CP*, *FGA*, *FGB*, and *FGG*), cathelicidins (*BAC5*, *BAC7.5*, *BAC3.4*, *MAP28*, and *MAP34*), defensins (*GBD1*, *beta-defensin-2*, and *HEPC*), and cytolytic protein (*LIZ* and *LF*) genes in the livers of young castrated bucks of the Polish White Improved (PWI) breed.

## 2. Materials and Methods

The research was carried out on 20 young Polish White Improved (PWI) bucks castrated at the age of 3 months. Only bucks free from small ruminant lentivirus (SRLV) infection were selected for the experiment, which was investigated on the basis of ELISA tests (ID Screen^®^ MVV-CAEV, Indirect Screening ELISA, IDvet Innovative Diagnostics, Grabels, France) following the manufacturer’s instructions. All animals in this herd had been under immunoassay control for more than 20 years as a part of another large-scale project dedicated to eradicating caprine arthritis encephalitis (CAE) from the herd [20]. ELISA tests were also conducted at the beginning and at the end of the experiment to confirm that no animal seroconverted during the study. The experiment began when the castrated bucks were 8 months old, had an average live weight of 28.80 kg (±4.93 kg), and lasted for 124 days. All animals were kept in the same conditions. They were under constant veterinary care and showed no signs of any disease. They were fed in a group system ad libitum, using good-quality hay, oat grains, wheat bran, and a vitamin and mineral mixture (Vitamix C, Polmass, Bydgoszcz, Poland) routinely mixed with concentrated feed, and had free and constant access to water and salt licks. The amount of forage fed per group covered the average life needs of the bucks, in accordance with the INRA system standards adapted to the nutritional value of feed used in Poland. The castrated bucks were divided into two groups: control (N = 10) and experimental (N = 10). The experimental group was administered supplement in the form of a mixture of dried extracts (curcumin to rosemary in a ratio of 896:19) from *C. longa* and *R. officinalis* (Selko^®^ AOmix; Amersfoort, The Netherlands)). The mixture is distributed in Poland by Trouw Nutrition Polska sp. z o.o. (Grodzisk Mazowiecki, Poland), a part of Dutch Nutreco company (Amersfoort, The Netherlands). The mixture is a commercial supplement used in the feed for dairy cows. Moreover, two other mixtures with the same trade names but different contents (direct information from producer) are dedicated to pigs and poultry. According to the producer, vitamin E can be partially replaced by this mixture thanks to the activity of its polyphenols as antioxidants and the fact that it supports productivity and the immune system and prevents lipid oxidation (https://www.trouwnutrition.com/en/programme-lister/ao-mix-170937/ (accessed on 27 September 2023)). The established dose recommended by the producer (1.6 g/day) for adult dairy goats, confirmed by its expert for young castrated bucks with live body mass of approx. 30 kg, was administered individually into the mouth in starch capsules before the morning feeding by animal handler each day of the 124-day trial. This person supervised whether the capsule was swallowed by the goats. During slaughter in the certified slaughterhouse, liver fragments were collected, immediately frozen in liquid nitrogen, and stored at −80 °C for further analysis.

Total RNA was isolated according to the producer protocol attached to RNeasy Mini Kit (Qiagen, Hilden, Germany), and then reverse transcription was performed using the Transcriptor First Strand cDNA Synthesis Kit (Roche, Basel, Switzerland). Isolated cDNA was diluted to 50 nq/µL and used as a matrix to perform quantitative reverse transcription PCR (RT-qPCR) using LightCycler^®^ 480 (Roche, Basel, Switzerland). Table S1 contains the primer sequences of the studied genes as well as the reference genes (cyclophilin A, *PPIA*; battenin, *CLN3*), which were selected from the set of three genes. The stability of *PPIA*, *CLN3*, and Glyceraldehyde-3-Phosphate Dehydrogenase (*GAPDH*) was analyzed using RefFinder (http://blooge.cn/RefFinder/ (accessed on 27 September 2023)) (Supplementary File S1). Sequences of gene primers were selected on the basis of literature data or were self-designed using the PRIMER3 program. The reaction was carried out in duplicate according to the producer’s protocol of the commercial LightCycler^®^ 480 SYBR Green I Master kit (Roche, Basel, Switzerland). The primer annealing temperature was checked during the preliminary tests performed on cDNA mixtures by PCR reaction in a temperature gradient, and the best temperature was found to be uniform for all primers used. The amplification process started with a pre-incubation at 95 °C for 5 min and then ran for 45 cycles in conditions as follows: denaturation at 95 °C for 5 s, then annealing at 60 °C for 15 s, and elongation at 72 °C for 20 s. The final elongation lasted 10 min at 72 °C. We used both positive and negative controls for the reaction. We used pooled cDNA of a few randomly selected samples as the positive control, while the mix without any cDNA was used as the negative control. To check the size, 2% agarose gel electrophoresis was used. Ethidium bromide was used to stain the gel. The size of the product was visualized under UV light using a G: BOX device (Syngene, Frederick, MD, USA).

To assess the normality of distribution, the PROCC UNIVARIATE of SAS package (SAS/STAT, 2002–2012, version 9.4) was used, and before the statistical analysis, the normalized expression levels of the studied genes were transformed using the natural logarithm scale. The analysis of variance was carried out using the GLM procedure of the SAS package with a model taking into account the fixed effect of the group with *t*-Student test as post hoc.

The following cut-off points for significance were chosen: the means differed significantly at *p* ≤ 0.05, and the values of *p* between 0.05 and 0.1 were considered to be at the trend level.

## 3. Results

**Figure 1:**
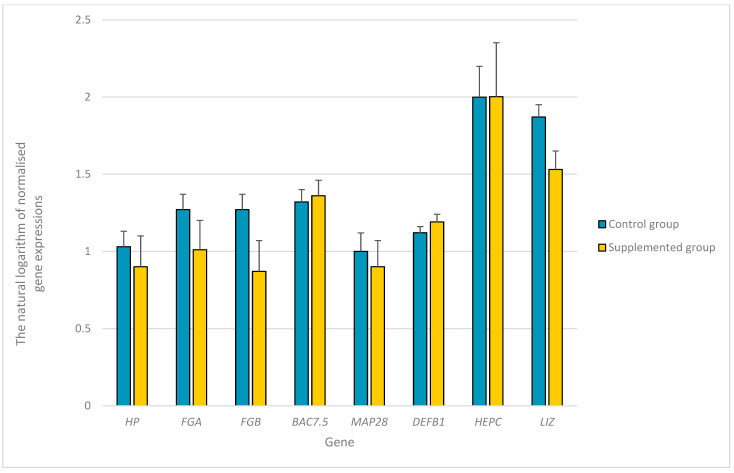
The natural logarithm of normalized expression of the selected genes with their standard errors in the livers of young castrated bucks on the diet supplemented with the mixture of dried extracts *C. longa* and *R. officinalis* (experimental group) and bucks on the diet without any supplementation (control group). *HP*—haptoglobin; *FGA*—fibrinogen α chain; *FGB*—fibrinogen β chain; *BAC7.5*—bactenecin-7.5; cathelicidin-3; *MAP28*—cathelicidin-6; *GBD1*—β-defensin 1; *HEPC*—hepcidin; *LIZ*—lysozyme.

The expression levels of *SAA*, *CRP*, *LALBA*, *AGP*, *CP*, *ChBac3.4*, *MAP34*, and beta-defensin-2 genes were below the detection limits. No differences were found between castrated bucks from the control and experimental groups in terms of the expression of *HP*, *FGA*, *FGB*, *BAC7.5*, *MAP28*, *GBD1*, *HEPC*, and *LIZ* in the livers of the castrated bucks (Figure 1).

**Figure 2:**
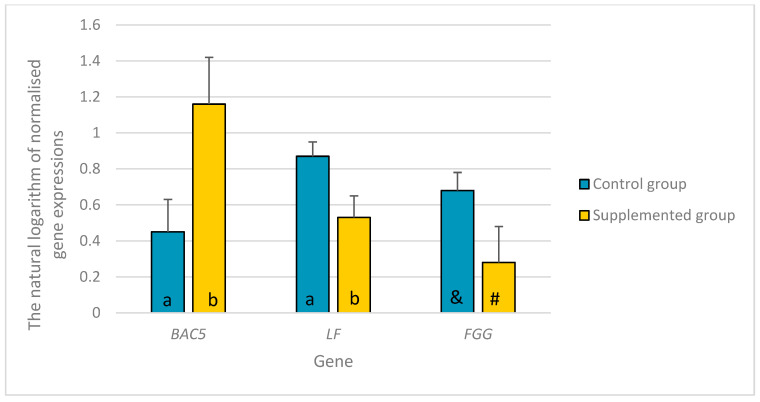
The differences in the natural logarithm of normalized expressions of the *BAC5*, *LF*, and *FGG* genes with their standard errors in livers between young castrated bucks on the diet a mixture of dried extracts of *C. longa* and *R. officinalis* supplementation (experimental group) and bucks on the diet without any supplementation (control group). a, b—different letters indicate the significance of differences at *p* ≤ 0.05; &, #—different symbols indicate the significance of differences at 0.05 < *p* ≤ 0.1; *BAC5*—bactenecin-5, cathelicidin 2; *LF*—lactoferrin; *FGG*—fibrinogen γ chain.

Lower *LF* and higher *BAC5* expression levels were recorded in the experimental group compared to those in the control group (*p* ≤ 0.05). A tendency was also found for the lower expression of *FGG* (*p* = 0.0723) in the experimental than in the control group (Figure 2).

## 4. Discussion

Of the nine APP genes we studied, we did not find any expression of five, namely *SAA*, *CRP*, *LALBA*, *AGP*, and *CP*, in the livers of the healthy young castrated bucks. As we stated the expression of reference genes in all samples and used the negative and positive controls in RT-qPCR analyses, we assume that their transcript levels were below the detection limit. To the best of our knowledge, most of these proteins are produced in the livers of ruminants or were found to be expressed in hepatocytes [21]. Therefore, the lack of their expression in our study may mean that their expression was very low in the livers of the bucks. The lack of expression of some APPs in the experimental and control groups or the lack of differences in their expression between the two groups indicates that there was no acute inflammation in the livers of animals in both groups. Moreover, it proves that the bucks used in our experiment were provided with a properly balanced diet. The spice–herbal mixture did not negatively affect the functioning of the liver.

The concentration of defensins in healthy tissue is usually low, and they are activated immediately after infection [22]. They contribute to the regulation of the innate immune response, and they also directly kill pathogens. So far, forty-eight goat beta-defensin genes and two pseudogenes in chromosomes 8, 13, and 23 have been identified [23]. Goat beta-defensin 124 (gDB-124) was found in many tissues and organs, including the liver [24]. However, the most studied defensins are *GBD1* and beta-defensin-2. *GBD1* has been found in the trachea, lungs, bronchi, and tongue, while beta-defensin-2 transcripts have been found in the digestive tract [25] as well as in the semen and blood of local Indian goat breeds [26]. On the one hand, Reczyńska et al. did not detect *GBD2* transcripts in the milk somatic cells (MSCs) of healthy dairy goats [27]. On the other hand, Zalewska et al. [12] and Jarczak et al. [28] did not detect *GBD1* but did detect beta defensin-2 at, respectively, the mRNA and protein levels in the MSCs of healthy goats. Moreover, Zalewska et al. [12] did not find any impact of organic selenium or rosemary–turmeric mixture (Selko^®^ AOmix, Trouw Nutrition Polska sp. z o.o., Grodzisk Mazowiecki, Poland) supplementation on the beta-defensin-2 content in the milk of healthy dairy goats. In our study, we did not find beta-defensin-2 in the liver of healthy bucks, but we detected *GBD1*, although without any difference between the two groups. This indicates that GBD1 may play an important role in the first line of immune response in the liver, whereas beta-defensin-2 is more important in maintaining homeostasis of the mammary gland.

LF has an immune role similar to that of HEPC and LIZ, but we only found differences in the expression levels of the *LF* gene in the present study. Some researchers indicate the therapeutic potential of LF in different types of liver diseases. Moreover, as LF is able to bind to many receptors on hepatocytes, its use as a targeting system to deliver drugs to the liver is promising [29]. The lack of differences between the control and experimental groups of castrated bucks in terms of the expression of the *LIZ* and *HEPC* genes proves that the animals of both groups were healthy. However, the lower expression of the *LF* gene, which as a protein product plays a similar role to that of HEPC and LIZ, in the experimental group of bucks suggests that supplementation with the mixture of dried extracts from *C. longa* and *R. officinalis* inhibits the activity of this protein.

We found the expression levels of only positive APPs in the livers of castrated bucks of both groups, and the expression of the *HP*, *FGA*, and *FGB* genes was unaltered. The production of Hp is stimulated by interleukin-1, interleukin-6, and TNF-α, which are proinflammatory cytokines [30]. Primarily, Hp binds to hemoglobin and heme and prevents the realising of iron. Moreover, it suppresses proinflammatory mediators, preventing bacterial proliferation. A higher concentration of Hp is a marker of the presence and severity of a few diseases. However, it is unknown whether a higher concentration of Hp has antibacterial and antiviral functions during the course of the disease [31].

Fb is a soluble glycoprotein composed of three polypeptide chains (alpha, beta, and gamma). Fb plays an essential role in homeostasis by participating in coagulation. In our study, the level of *FGG* transcripts decreased in the liver of the castrated bucks that were administered a mixture of dried extracts from *R. officinalis* and *C. longa*, while high levels of *FGB* and *FGG* were found in cattle liver, wherein their expression increased during endometritis. The downregulation of *FGG* expression in the liver of the experimental animals suggests the possible inhibition of the start of the signaling cascade [11]. Thus, the results prove that the castrated bucks in both groups did not have disturbed homeostasis.

There were no differences in the expression levels of two of the five cathelicidins, namely *BAC7.5* and *MAP28* between groups, which may indicate that they are natural elements of hepatic immune defense and evidence of the good health of the bucks in both groups. The expression level of only one of the five cathelicidins, *BAC5*, was higher in the experimental group than in the control group. In mammals, cathelicidins occur in immune cells (neutrophils), the cells of lymphatic organs (bone marrow), the liver and intestines [32], mammary gland tissue, and the teat of goats [33]. BAC7.5 exhibits significant antimicrobial activity against Gram-negative and Gram-positive bacteria by inactivating them without damaging their membranes [34]. If homeostasis is not disturbed, the activity of these potent effector molecules is not essential. Similarly, MAP28 participates in the innate immune system of goats. It exhibits antimicrobial, antiviral, and antifungal activity. Isobe [35] suggests that the secretion of cathelicidins in milk is stimulated by bacteria. However, different types of cathelicidins are produced by different cells in mammary glands. These results (although they refer to milk) were partially confirmed in our study: not all studied cathelicidins are produced by liver cells. For the first time, we proved the expression of cathelicidin genes in goat liver. It seems that BAC5 is important in liver functioning and homeostasis balance and, therefore, responds to supplementation. It is possible that the supplement mixture helps in maintaining homeostasis, thereby increasing BAC5 expression.

## 5. Conclusions

The lack of expression of some of the studied genes and the lack of differences in the expression levels of some between the control and experimental groups of bucks indicate that the animals in both groups were healthy, had no acute inflammation, and had no homeostasis disturbances. Thus, they were fed properly, and their welfare was preserved. The higher expression of *LF* in the control group suggests that it may be important for the first line of hepatic immune defense and its expression is downregulated by the mixture of turmeric and rosemary extracts. In other words, the spice–herb mixture mutes LF activity. As LF has therapeutic potential during the course of different types of liver diseases, it may be concluded that the supplementation used in the study, which lowered the expression of *LF* in the liver, negatively influences the liver’s resistance to some diseases. Therefore, the question arises as to whether the mixture dose used was too high. Another study is needed with different levels of supplementation in order to investigate this issue. The lower expression of *FGB* and the higher expression of *BAC5* genes in the livers of the castrated bucks that were administered the supplement suggest the silencing effects of the mixture on the acute immune response as well as its stimulating effect on antimicrobial immunity. Because the expression of both APPs and cathelicidins is related to the expression of cytokines, in order to fully assess the effect of supplementation with a mixture of dry rosemary and turmeric extracts, the expression of the cytokine gene should be investigated.

## Data Availability

The datasets used and/or analyzed during the current study are available from the corresponding author on reasonable request.

## Supplementary Materials

The following supporting information can be downloaded at: https://www.mdpi.com/article/10.3390/genes14101932/s1, Figure S1. Comprehensive gene stability. (*PPIA*-cyclophilin A, *CLN3*-battenin, and *GAPDH*-Glyceraldehyde-3-Phosphate Dehydrogenase). Figure S2. Gene stability by Delta CT method. (*PPIA*-cyclophilin A, *CLN3*-battenin, and *GAPDH*-Glyceraldehyde-3-Phosphate Dehydrogenase). Figure S3. Gene stability by BestKeeper with Pearson correlation coefficient (r) BestKeeper vs. selected gene. Figure S4. Gene stability by normFinder. Figure S5. Gene stability by Genorm. Table S1. Gene name, gene symbol, primer sequences, amplification product size, gene accession number in GenBank, and reference for primer sequences [16,21,28,33,36,37,38,39].

## References

[1] Zuliani A., Esbjerg L., Grunert K.G., Bovolenta S. (2018). Animal Welfare and Mountain Products from Traditional Dairy Farms: How Do Consumers Perceive Complexity?. Animals.

[2] Stanger B.Z. (2015). Cellular Homeostasis and Repair in the Mammalian Liver. Annu. Rev. Physiol..

[3] Kubes P., Jenne C. (2018). Immune Responses in the Liver. Annu. Rev. Immunol..

[4] Zhang H., Tsao R. (2016). Dietary polyphenols, oxidative stress and antioxidant and anti-inflammatory effects. Curr. Opin. Food Sci..

[5] Qadir M.I., Qousain S.T., Muhammad S.A. (2016). Curcumin: A Polyphenol with Moleculat Targets for Cancer Control. Asian Pac. J. Cancer Prev..

[6] Domarla S.R., Komma R., Bhatnagar U., Rajesh N., Mulla S.M.A. (2018). An Evaluation of the Genotoxicity and Subchronic Oral Toxicity of Synthetic Curcumin. J. Toxicol..

[7] Petiwala S.M., Puthenveetil A.G., Johnson J.J. (2013). Polyphenols from the Mediterranean herb rosemary (*Rosmarinus officinalis*) for prostate cancer. Front. Pharmacol..

[8] Murata H., Shimada N., Yoshioka M. (2004). Current research on acute phase proteins in veterinary diagnosis: An overview. Vet. J..

[9] Ceciliani F., Ceron J.J., Eckersall P.D., Sauerwein H. (2012). Acute phase proteins in ruminants. J. Proteom..

[10] Mookherjee N., Anderson M.A., Haagsman H.P., Davidson D.J. (2020). Antimicrobial host defence peptides: Functions and clinical potential. Nat. Rev. Drug Discov..

[11] Reczyńska D., Zalewska M., Czopowicz M., Kaba J., Zwierzchowski L., Bagnicka E. (2018). Acute Phase Protein Levels as An Auxiliary Tool in Diagnosing Viral Diseases in Ruminants—A Review. Viruses.

[12] Zalewska M., Kapusta A., Kawecka-Grochocka E., Urbańska D.M., Czopowicz M., Kaba J., Brzozowska P., Bagnicka E. (2022). Effect of Supplementation with Organic Selenium or Turmeric and Rosemary Mixture on Beta-Defensin Content in Goat Milk. Animals.

[13] Zhang G., Wu H., Shi J., Ganz T., Ross C.R., Blecha F. (1998). Molecular cloning and tissue expression of porcine beta-defensin-1. FEBS Lett..

[14] Sevgisunar N.S., Şahinduran S. (2021). Evaluation of Some Ecute Phase Proteins, Cytokines and Hepcidin Levels in Naturally Infected Saanen Goats with Paratuberculosis. MAKU J. Health Sci. Inst..

[15] Michels K., Nemeth E., Ganz T., Mehrad B. (2015). Hepcidin and Host Defense against Infectious Diseases. PLoS Pathog..

[16] Shamova O., Brogden K.A., Zhao C., Nguyen T., Korkyakov V.N., Lehrer R.I. (1999). Purfication and Properties of Proline-Rich Antimicrobial Peptides from Sheep and Goat Leukocytes. Infect. Immun..

[17] Aksel E.G., Akyüz B. (2021). Effect of LPS and LTA stimulation on the expression of TLR-pathway genes in PBMCs of *Akkaraman lambs* in vivo. Trop. Anim. Health Prod..

[18] Giansanti F., Panella G., Leboffe L., Antonini G. (2016). Lactoferrin from milk: Nutraceutical and pharmacological properties. Pharmaceuticalis.

[19] Ferraboschi P., Ciceri S., Grisenti P. (2021). Applications of Lysozyme, an Innate Immune Defense Factor, as an Alternative Antibiotic. Antibiotics.

[20] Czopowicz M., Szaluś-Jordanow O., Moroz A., Mickiewicz M., Witkowski L., Markowska-Daniel I., Bagnicka E., Kaba J. (2018). Use of two commercial caprine arthritis–encephalitis immunoenzymatic assays for screening of arthritic goats. J. Vet. Diagn. Investig..

[21] Dong H., Wang S., Jia Y., Ni Y., Zhang Y., Zhuang S., Shen X., Zhao R. (2013). Long-Term Effects of Subacute Ruminal Acidosis (SARA)on Milk Quality and Hepatic Gene Expression in Lactating Goats Fed a High-Concentrate Diet. PLoS ONE.

[22] Wilson S.S., Wiens M.E., Smith J.G. (2013). Antiviral mechanisms of human defensins. J. Mol. Biol..

[23] Zhang L., Xiao H., Huang J., Ouyang L., Li S., Tang Y. (2021). Identification and expression analysis of the β-defensin genes in the goat small intestine. Gene.

[24] Tai M., Jiang Y., Ren Y., Jin L., Qiao L., Zhang C. (2017). Expression pattern analysis of goat beta-defensin 124 and its location in the reproductive organs. Acta Vet. Zootech. Sin..

[25] Bagnicka E., Prusak B., Kościuczuk E., Jarczak J., Kaba J., Strzałkowska N., Jóźwik A., Czopowicz M., Krzyżewski J., Zwierzchowski L. (2013). A Note on the Organization and Expression of β-Defensin Genes in Polish Goats. J. Appl. Genet..

[26] Ranjan R., Singh P., Singh S.P., Gururaj K., Kharche S.D., Singh M.K. (2021). Status of Beta Defensin-1 and Its Effect on Post Thaw Semen Fertility Gene Expression in Indian Goat Breed. Cryoletters.

[27] Reczyńska D., Witek B., Jarczak J., Czopowicz M., Mickiewicz M., Kaba J., Zwierzchowski L., Bagnicka E. (2019). The Impact of Organic vs. Inorganic Selenium on Dairy Goat Productivity and Expression of Selected Genes in Milk Somatic Cells. J. Dairy Res..

[28] Jarczak J., Kościuczuk E., Ostrowska M., Lisowski P., Strzałkowska N., Jóźwik A., Krzyżewski J., Zwierzchowski L., Słoniewska D., Bagnicka E. (2014). The effects of diet supplementation with yeast on the expression of selected immune system genes in the milk somatic cells of dairy goats. Anim. Sci. Pap. Rep..

[29] Hessin A., Hegazy R., Hassan A., Yassin N., Kenawy S. (2015). Lactoferrin enhanced apoptosis and protected against thioacetamide-induced liver fibrosis in rats. Open Access Maced. J. Med. Sci..

[30] Popko K., Gorska E., Stelmaszczyk-Emmel A., Plywaczewski R., Stoklosa A., Gorecka D., Pyrzak B., Demkow U. (2010). Proinflammatory cytokines Il-6 and TNF-α and the development of inflammation in obese subjects. Eur. J. Med. Res..

[31] Reczyńska D., Zalewska M., Czopowicz M., Kaba J., Zwierzchowski L., Bagnicka E. (2018). Small ruminant lentivirus infection influences expression of acute phase proteins and cathelicidin genes in milk somatic cells and peripheral blood leukocytes of dairy goats. Vet. Res..

[32] Deptuła J., Tokarz-Deptuła B., Malinowska-Borysiak M., Stosik M., Deptuła W. (2019). Cathelicidins in humans and animals. Adv. Microbiol. Postępy Mikrobiol..

[33] Zhang G.-W., Lai S.-J., Yoshimura Y., Isobe N. (2014). Expression of cathelicidins mRNA in the goat mammary gland and effect of the intramammary infusion of lipopolysaccharide on milk cathelicidin-2 concentration. Vet. Microbiol..

[34] Shamova O.V., Orlov D.S., Zharkova M.S., Balandin S.V., Yamschikova E.V., Knappe D., Hoffmann R., Kokryakov V.N., Ovchinnikova T.V. (2016). Minibactenecins ChBac7.Nα and ChBac7. Nβ—Antimicrobial Peptides from Leukocytes of the Goat Capra hircus. Acta Naturae.

[35] Isobe N. (2017). Control mechanisms for producing antimicrobial factors in ruminant mammary gland. Anim. Sci. J..

[36] Jarczak J., Kaba J., Bagnicka E. (2014). The validation of housekeeping genes as a reference in quantitative Real Time PCR analysis. Application in the milk somatic cells and frozen whole blood of goats infected with caprine arthritis encephalitis virus. Gene.

[37] Brenaut P., Lefèvre L., Rau A., Laloë D., Pisoni G., Moroni P., Bevilacqua C., Martin P. (2014). Contribution of mammary epithelial cells to the immune response during early stages of a bacterial infection to *Staphylococcus aureus*. BMC Vet. Res..

[38] Ceciliani F., Rahman M.M., Lecchi C., Maccalli M., Pisoni G., Sartorelli P. (2009). Systemic and in vitro expression of goat α-acid glycoprotein during Caprine Arthritis-Encephalitis Virus infection. Vet. Immunol. Immunopathol..

[39] Restelli L., Codrea M.C., Savoini G., Ceciliani F., Bendixen E. (2014). LC-MS/MS analysis of visceral and subcutaneous adipose tissue proteomes in young goats with focus on innate immunity and inflammation related proteins. J. Proteom..

