# Supplementary material for: Effect of Supplementation with Curcuma longa and Rosmarinus officinalis Extract Mixture on Acute Phase Protein, Cathelicidin, Defensin and Cytolytic Protein Gene Expression in the Livers of Young Castrated Polish White Improved Bucks
Source: Genes (Basel). 2023 Oct 12;14(10):1932. doi: 10.3390/genes14101932 (PMC10606746; doi:10.3390/genes14101932)
Supplement: Supplementary file 1 [file genes-14-01932-s001.zip › genes-2590137-supplementary.pdf]

Table S1. Gene name, gene symbol, primer sequences, amplification product size, gene accession number in GenBank, and reference for primer sequences.

Group of genes	Gene name	Gene symbol	Primer sequences		Product size (bp*)	GenBank^ /UniProt Accession	Reference
References	Cyclophilin A	*PPIA*	F	GGATTTATGTGCCAGGGTGGTGA	120	AY_247029.1	[36]
			R	CAAGATGCCAGGACCTGTATG			
	Battenin	*CLN3*	F	TTCTGACTCCTTGGGACACA	62	NM_001075174	[36]
			R	CAACCTGCCCACCTATCAGT			
3939Acute-phase proteins	Serum amyloid	*SAA*	F	CTGGGCTGCTAAAGTGATCAGTAAC	69	EU884570.1	[37]
			R	CCCTTGAGCAGAGGGTCTGTGATT			
	Haptoglobin	*HP*	F	TAATGCCCATCTGCCTAC	162	XM_005692202.3	[21]
			R	CGCCCTCATAGTGTTTCA			
	C–reactive protein	*CRP*	F	CTGGCTTGGGAGATTG	134	XM_018046353.1	[21]
			R	AGTGAGGGTAAGGGATT			
	α - lactalbumin	*LALBA*	F	TGACATTTGTGTGTGCCAAGA	198	NM_001285635.1	&PRIMER 3
			R	CAAGGGGGTACAAAGAAGCA			
	α - acid glycoprotein	*AGP*	F	TTGCTTGGCTGCAGGTGT	197	XM_012152252.2	[38]
			R	CAATGGTCTGGTACTCTCTCTG			
	Ceruloplasmin	*CP*	F	GAGCATGAAGGGGCCATTTATC	130	NM_001256556.1	[39]
			R	GCTGTCTTCCTCACCAGG			
	Fibrinogen α chain	*FGA*	F	TGAGATCCTGAGGCGCAAAG	104	NM_001033626.1	[39]

			R	TGTCCACCTCCA ATCGTTTCAT			
	Fibrinogen β chain	*FGB*	F	GACAACGACGG CTGGAAAAC	124	NM_00114 2917	[39]
			R	ACGCTCCACCCC AGTAGTAT			
	Fibrinogen γ chain	*FGG*	F	TGCCAATAAGG GGGCCAAAG	134	NM_17391 1	[39]
			R	GACTGCCATCA AGCCTCTTCT			
Catheli cidins	Bactenecin-5; cathelicidin-2	*BAC5*	F	GTGGAATTCAC GGTGAAGGAGA C	390	Y18873.1	[33]
			R	CTCAGGCCAAA TGAGAT			
	Bactenecin-7.5; cathelicidin-3	*BAC7.5*	F	GTGGAATTCAC GGTGAAGGAGA C	383	AJ243125. 1	[33]
			R	AGTGCTAACCTT GATGTT			
	Capra hircus bactinecin 3.4	*ChBac3. 4*	F	GCTAATCTCTAC CGCCTCCTGG	172	P80054	[16]
			R	CCACACACTGTT TCACCAGCC			
	Cathelicidin -6	*MAP28*	F	GTGGAATTCAC GGTGAAGGAGA C	225	AJ243126. 1	[33]
			R	AATTGGGCCGA CTTTGTGCC			
	Cathelicidin -7	*MAP34*	F	ACCGAATTCAG CTACAGGGAGG CCGT	428	AJ243127. 1	[33]
			R	ACCTGATCCTTA GGACTTC			
Defensi ns	β-defensin 1	*GBD1; DEFB1*	F	ACTCAAGGAAT AAGAAGTCG	112	Y17679	[39]
			R	CATTTTACTGGG GGCCCGAA			
	β-defensin 2	beta-defensin -2	F	ACTCAAGGAAT AATAAATCA	112	AJ009877	[39]
			R	CATTTTACTGGG GGCCCGTG			
	Hepcidin	*HEPC*	F	ACCTGCCTTCTGC TCCTTGT	103	GQ901053 .1	[28]

			R	CTCCAGCTGTGTG CTGAGTTT			
Cytolytic proteins	Lysozyme	*LIZ*	F	TGAAGGCTCTC ATTATTCTG	446	GQ889414.1	[28]
			R	TTACACTCCACA ACCCTG			
	Lactoferrin	*LF*	F	ACAGTAGCCTA GATTGTGTG	247	NC_FJ609300	&PRIMER 3
			R	CCTCATTTGCTT TCTTGAC			

*bp – base pairs F – forward primer R – reverse primer

Supplement file 1 – results of the ReFinder analyses

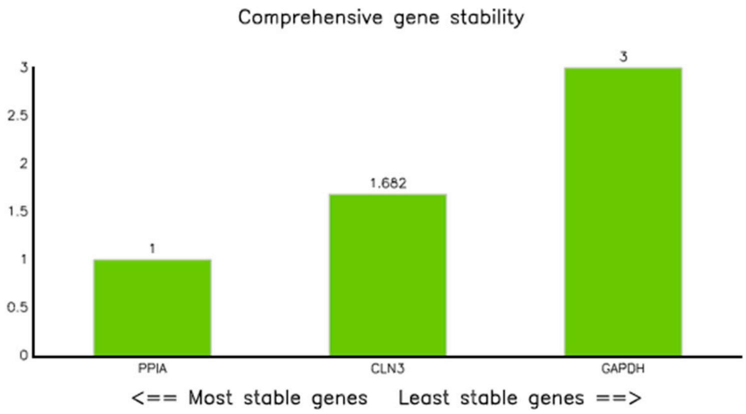

Figure S1. Comprehensive gene stability. (PPIA - cyclophilin A, CLN3 - battenin, and GAPDH - Glyceraldehyde-3-Phosphate Dehydrogenase).

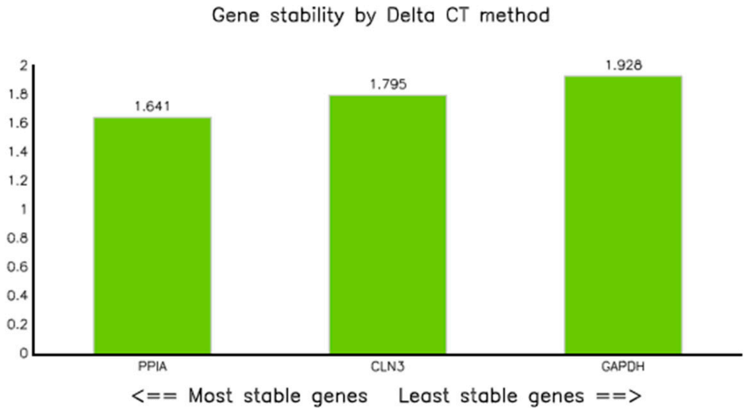

Figure S2. Gene stability by Delta CT method. (PPIA - cyclophilin A, CLN3 - battenin, and GAPDH - Glyceraldehyde-3-Phosphate Dehydrogenase).

CP data of housekeeping Genes by BEST KEEPER

	PPIA	CLN3	GAPDH
n	12	12	12
geo Mean [CP]	27.90	30.40	24.53
AR Mean [CP]	27.92	30.42	24.58
min [CP]	26.00	29.00	22.00
max [CP]	29.00	32.00	27.00
std dev [+/- CP]	0.78	0.82	1.25
CV [% CP]	2.79	2.69	5.08
min [x-fold]	-3.73	-2.64	-5.79
max [x-fold]	2.14	3.03	5.53
std dev [+/- x-fold]	1.71	1.76	2.38

Pearson correlation coefficient (r) by BEST KEEPER

	PPIA	CLN3	GAPDH
CLN3	-0.145	-	-
p-value	0.653	-	-
GAPDH	0.145	-0.220	-
p-value	0.652	0.491	-

Pearson correlation coefficient (r)

BestKeeper vs.	PPIA	CLN3	GAPDH
coeff. of corr. [r]	0.520	0.163	0.821
p-value	0.083	0.613	0.001

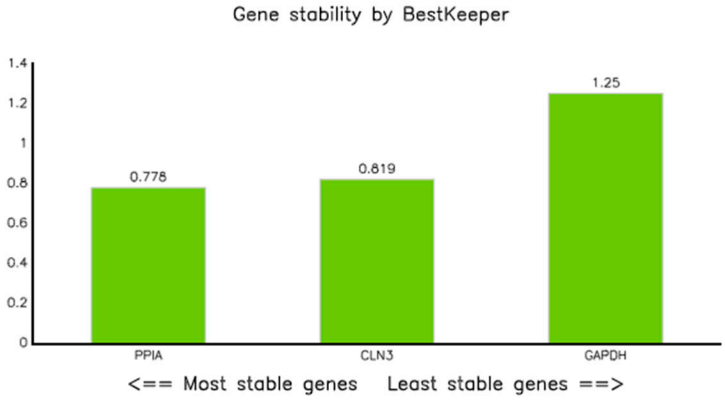

Figure S3. Gene stability by BestKeeper with Pearson correlation coefficient (r) BestKeeper vs. selected gene.

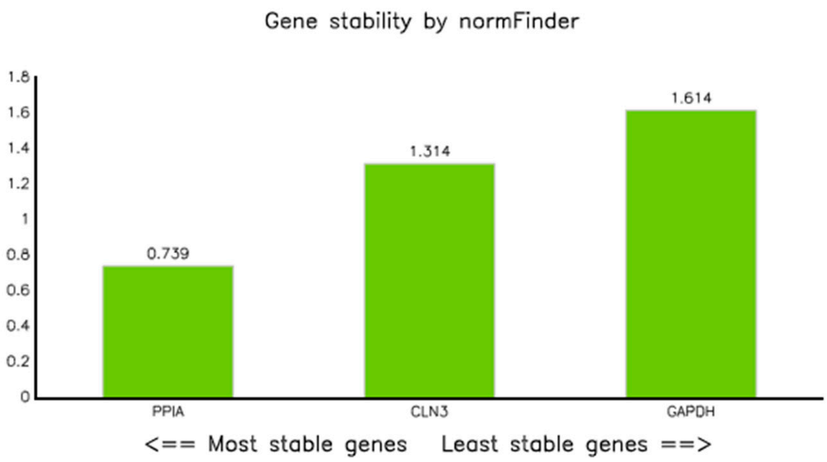

Figure S4. Gene stability by normFinder

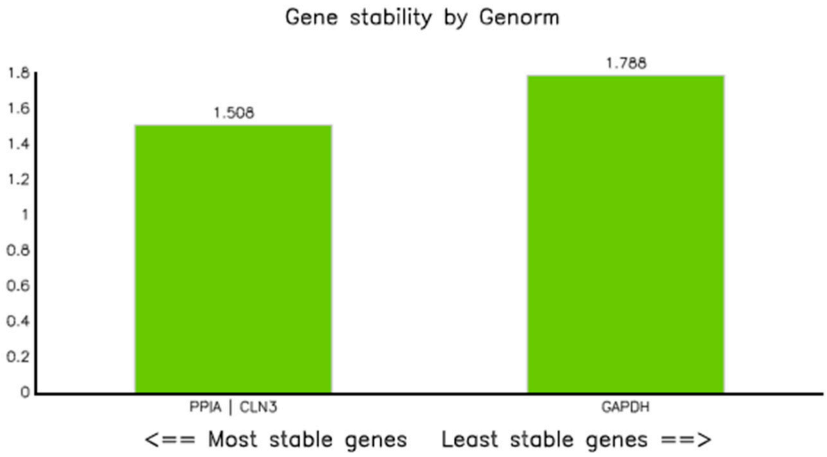

Figure S5. Gene stability by Genorm

[1]M = average expression stability, calculated by geNorm algorithm in the qBase software.
[2]Stability value = stability of gene expression calculated by intra and intergroup variation by NormFinder algorithm.
[3]Std dev [±CP] = standard deviation in BestKeeper algorithm, significant values < 1.0.
